# Supplementary material for: Profiling of intestinal T cells reveals antigen-specific dependency on early-life programming for the generation of microbiota-reactive Tregs
Source: bioRxiv. 2026 Sep 29:2026.09.23.753882. Preprint. [Version 1] doi: 10.64898/2026.09.23.753882 (PMC13648455; doi:10.64898/2026.09.23.753882)
Supplement: Supplementary file 1 [file NIHPP2026.09.23.753882v1-supplement-1.pdf]

bioRxiv preprint doi: https://doi.org/10.64898/2026.09.23.753882; this version posted September 29, 2026. The copyright holder for this preprint (which was not certified by peer review) is the author/funder, who has granted bioRxiv a license to display the preprint in perpetuity. It is made available under a CC-BY 4.0 International license.

SUPPLEMENTAL FIGURES

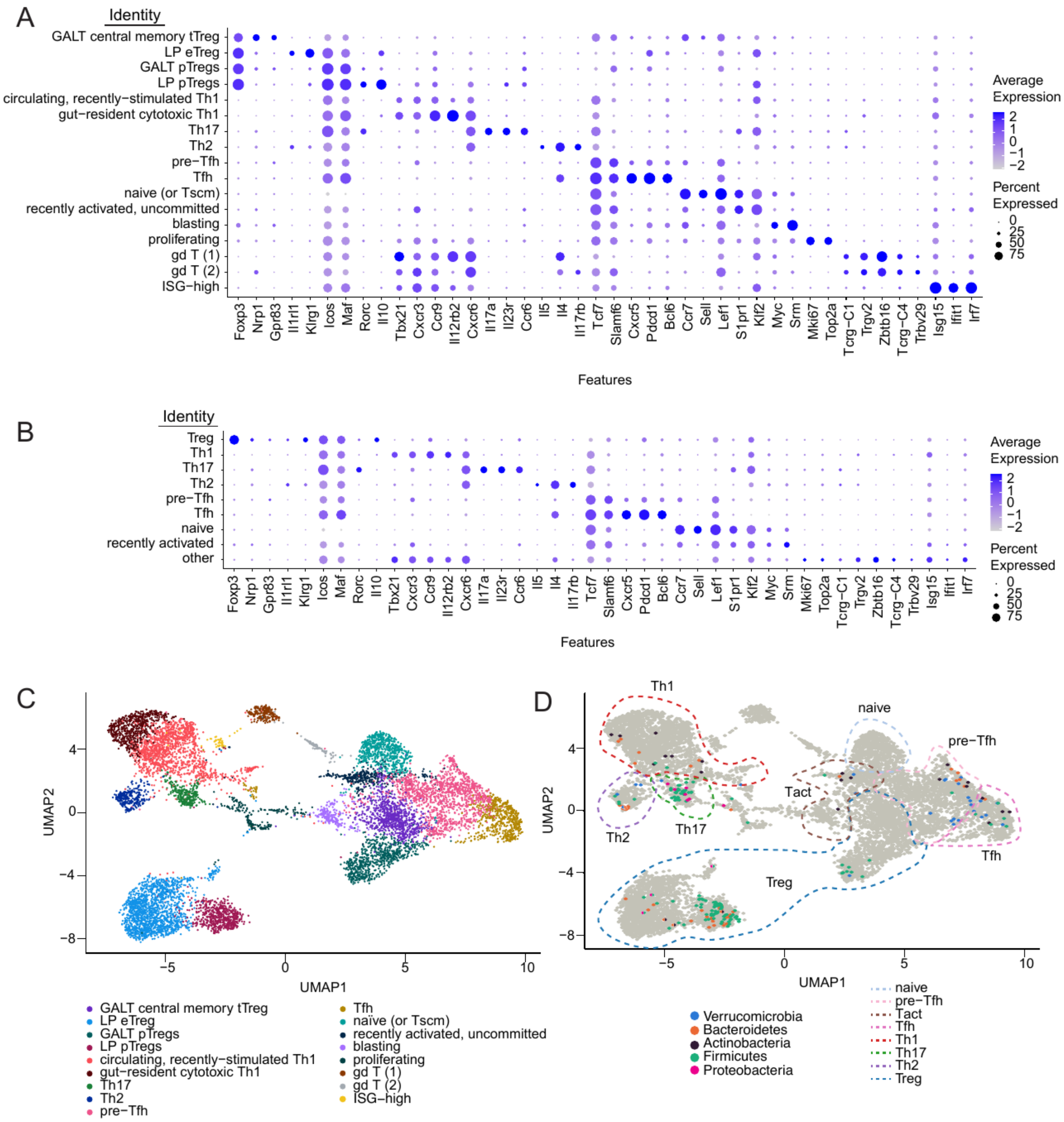

Figure S1. | Marker-gene validation of T cell phenotype calls for single cell clusters, related to Figure 1. (A) Dot plot of relevant gene expression used to define Th clusters shown in Figure S1D. (B) Dot plot of relevant gene expression in manually-merged, broad Th clusters related to Figure 1D-F. (C) UMAP of scRNA-seq cluster assignments based on key marker expression shown in S1A and described in Methods. Tscm = T stem-like central memory, ISG=Interferon stimulated genes. (D) UMAP showing location of phyla level specificity of cells expressing microbiota-reactive TCRs. Broadly categorized T cell clusters shown in 1D are indicated with dashed lines and labeled.

bioRxiv preprint doi: https://doi.org/10.64898/2026.09.23.753882; this version posted September 29, 2026. The copyright holder for this preprint (which was not certified by peer review) is the author/funder, who has granted bioRxiv a license to display the preprint in perpetuity. It is made available under a CC-BY 4.0 International license.

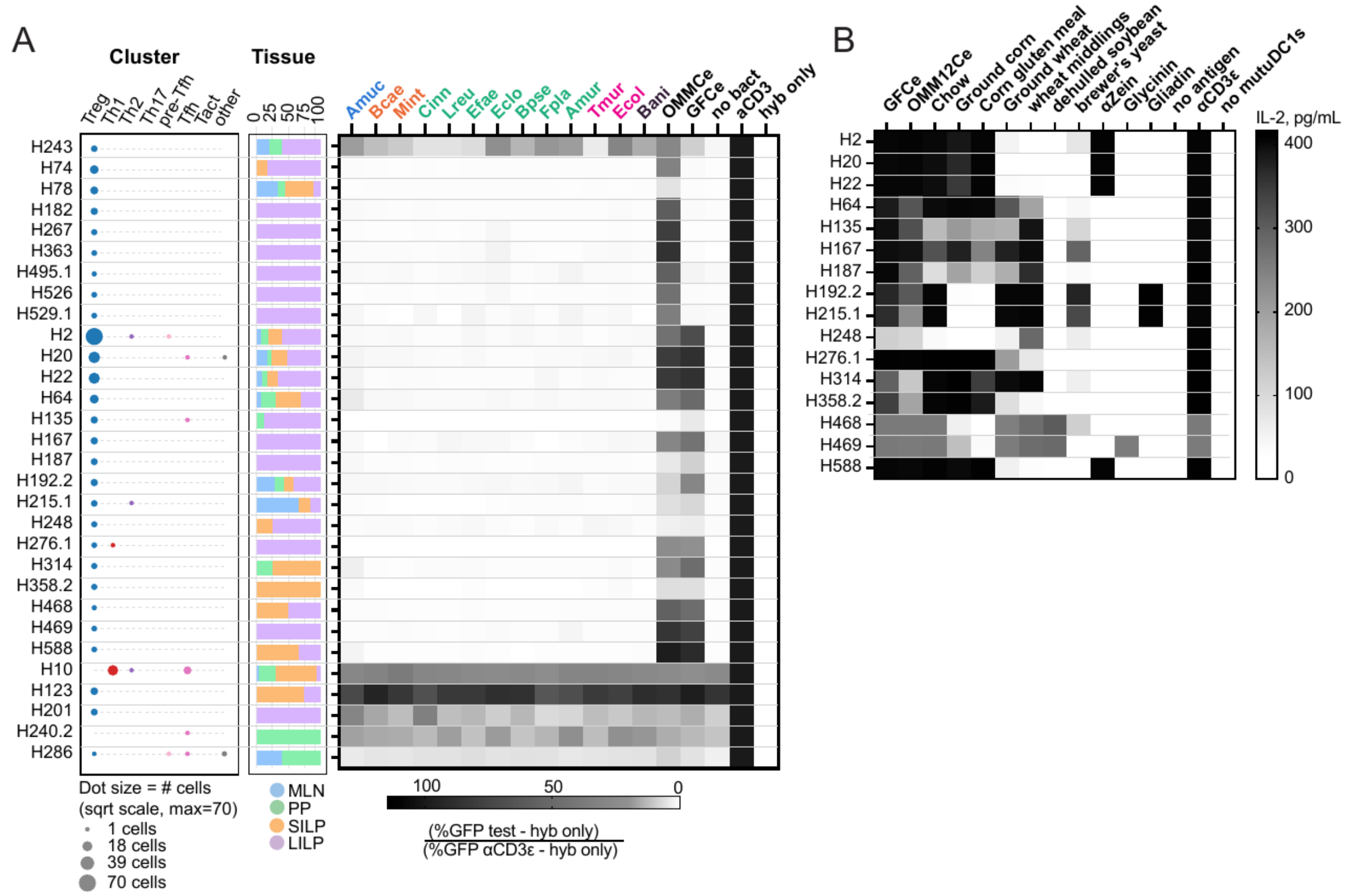

Figure S2. | Additional microbiota/dietary TCR reactivity screening, related to Figure 1. (A) Compilation of Th phenotype (left), tissue origin (middle) and reactivity (right) data for T cells with reactivity that could not be mapped to individual OMM12 members (OMMCe+ = microbiota-reactive but not mappable to a single microbe, GFCe+ and OMMCe+ = likely food or self). (B) Specificity of food-reactive TCRs to components of mouse chow or known food peptides. αZein peptide (FYQQPIIGGAL), Glycinin peptide (EYVSFKTNDT), gliadin peptide (CNVYIPPYCTIAP).

bioRxiv preprint doi: https://doi.org/10.64898/2026.09.23.753882; this version posted September 29, 2026. The copyright holder for this preprint (which was not certified by peer review) is the author/funder, who has granted bioRxiv a license to display the preprint in perpetuity. It is made available under a CC-BY 4.0 International license.

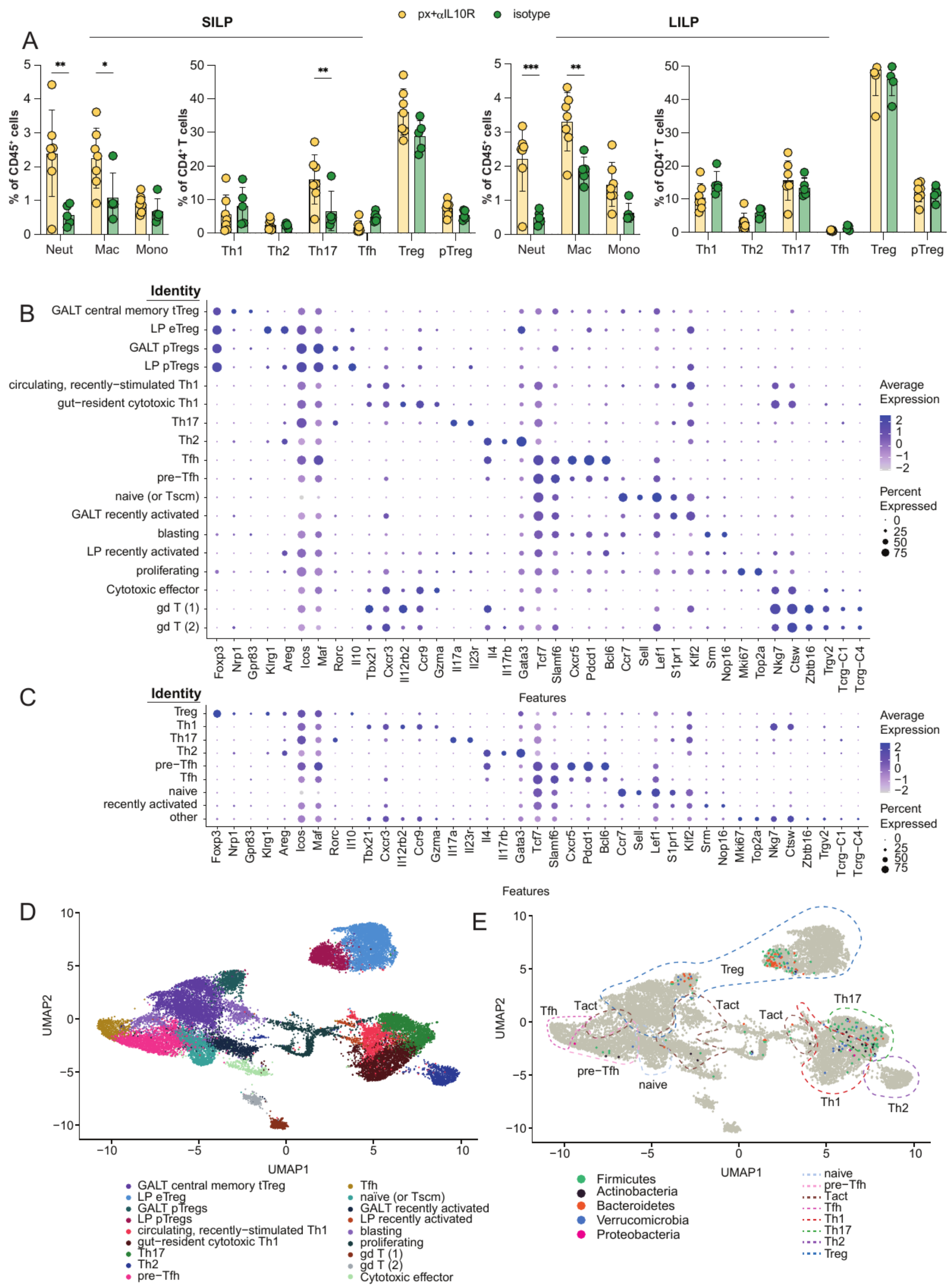

Figure S3. | Validation of intestinal inflammation and T cell phenotype clusters by marker gene expression, related to Figure 2. (A) Summary of the frequencies of myeloid (neutrophils(Neut), macrophages(mac), and monocytes(mono)) as well as T helper(Th) cells collected at d17 from the SILP (Left) and LILP (Right) of the indicated groups of mice. Statistical significance was determined by two-way ANOVA with Sidak's multiple comparisons test. *p < 0.05, **p < 0.01, ***p < 0.001. (B) Dot plot of relevant gene expression used to define Th clusters shown in Figure S3D. (C) Dot plot of relevant gene expression of manually-merged broad Th phenotype clusters related to Figure 2E. (D) UMAP of scRNA-seq cluster assignments based on key marker expression (described in Methods). (E) UMAP showing location of phyla level specificity of cells expressing microbiota-reactive TCRs. Broadly categorized T cell clusters shown in Figure 1B are indicated with dashed lines and labeled.

bioRxiv preprint doi: https://doi.org/10.64898/2026.09.23.753882; this version posted September 29, 2026. The copyright holder for this preprint (which was not certified by peer review) is the author/funder, who has granted bioRxiv a license to display the preprint in perpetuity. It is made available under a CC-BY 4.0 International license.

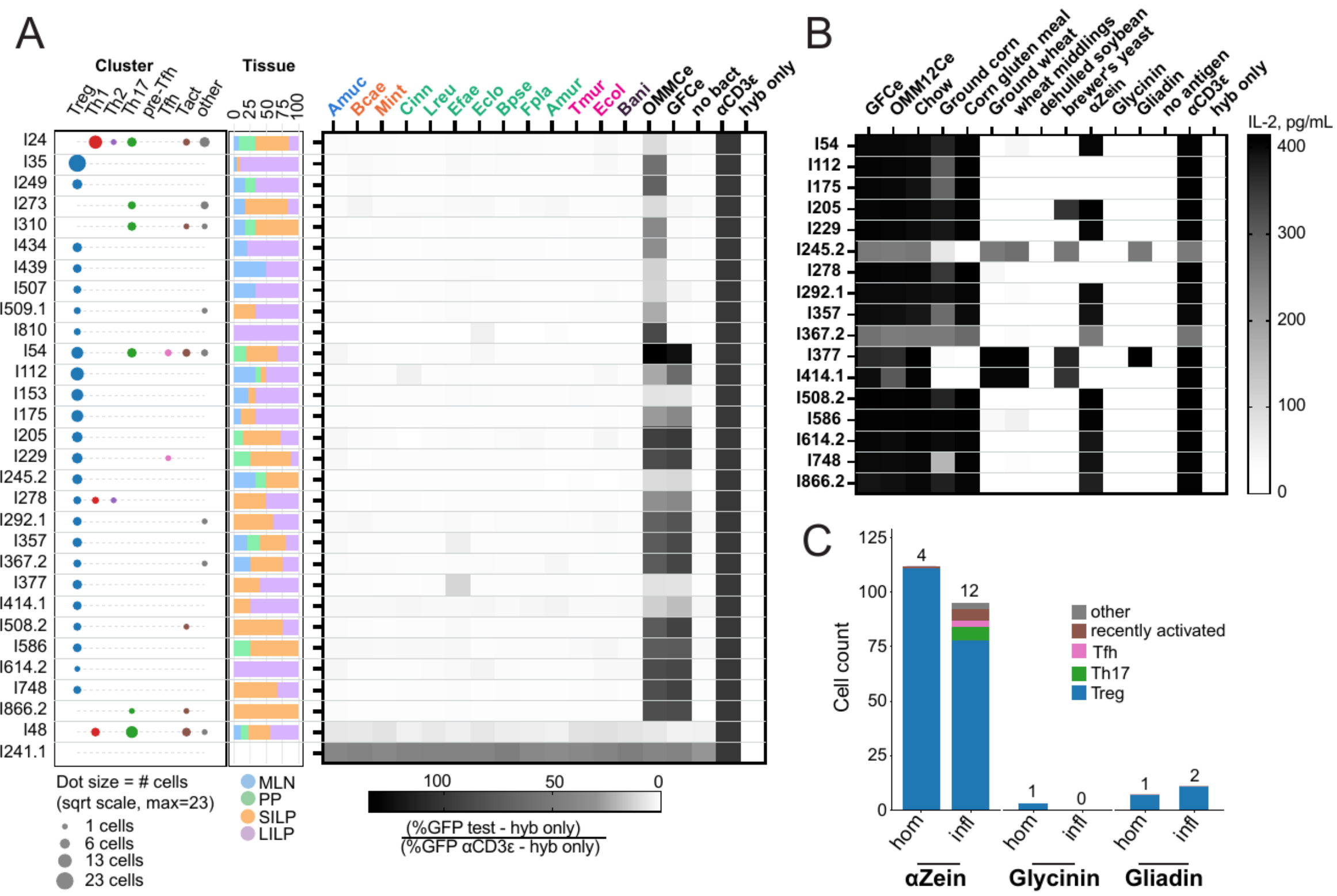

Figure S4. | Additional microbiota/dietary TCR reactivity screening, related to Figure 2. (A) Compilation of Th phenotype (left), tissue origin (middle) and reactivity (right) data for T cells with reactivity that could not be mapped to individual OMM12 members (OMMCe+ = microbiota-reactive but not mappable to a single microbe, GFCe+ and OMMCe+ = probably food, or self). (B) Specificity of food-reactive TCRs to components of mouse chow or known food peptides. (C) Phenotypes of cells present in the homeostasis and inflammation single cell datasets that recognize αZein, glycinin and gliadin peptides. Note: inflammation clonotype 17 (I17) was identical to homeostasis clonotype 2 and therefore I17 is not included in S4A and S4B but is included in the cell counts for αZein-reactive cells during inflammation.

bioRxiv preprint doi: https://doi.org/10.64898/2026.09.23.753882; this version posted September 29, 2026. The copyright holder for this preprint (which was not certified by peer review) is the author/funder, who has granted bioRxiv a license to display the preprint in perpetuity. It is made available under a CC-BY 4.0 International license.

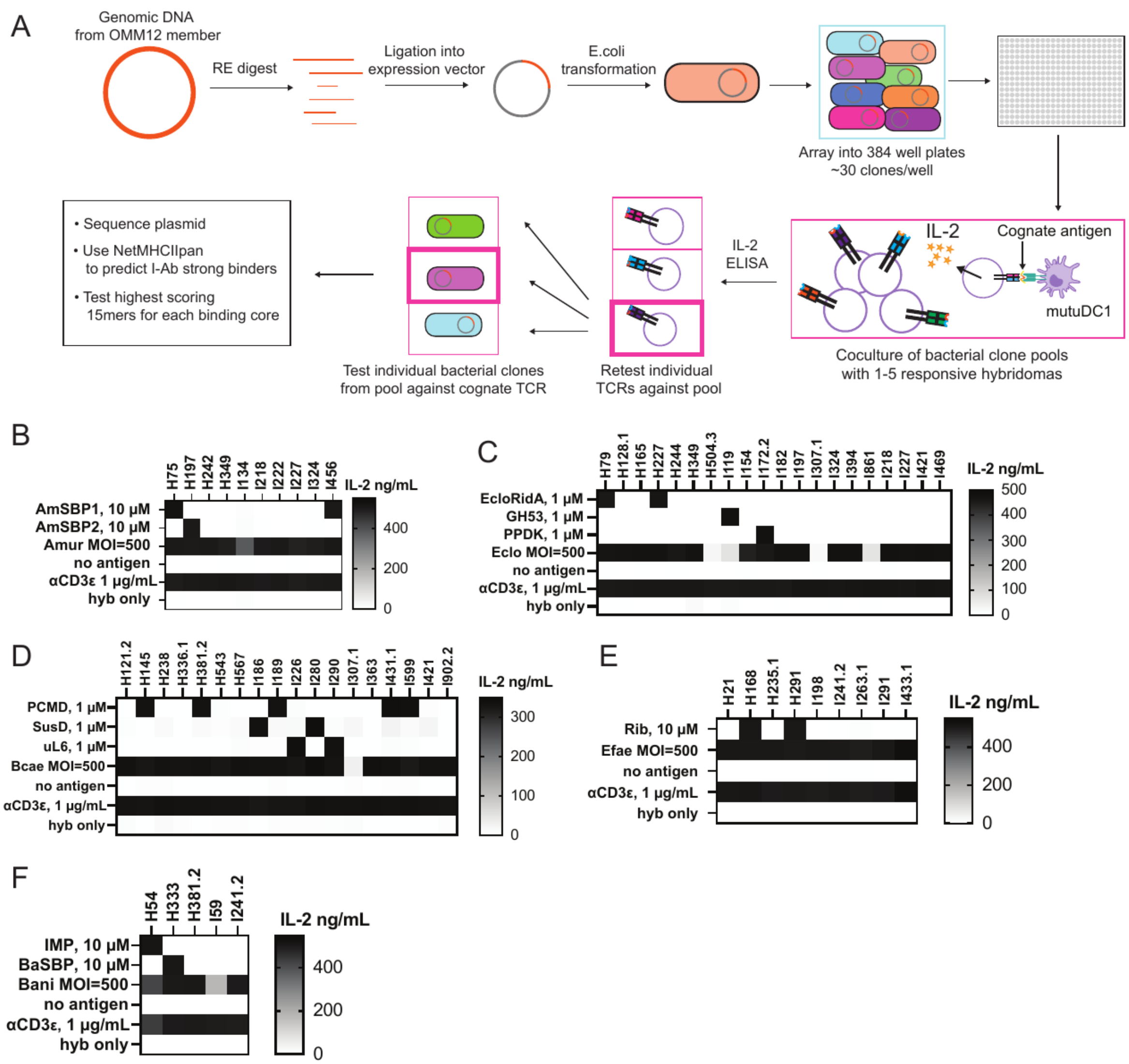

Figure S5. | Peptide discovery pipeline and validation of peptide stimulation of additional TCRs, related to Figure 3. (A) Overview of antigen discovery pipeline. Genomic DNA from one of the OMM12 microbes was isolated and partially digested to generate a range of fragment sizes. The DNA fragments were ligated into an IPTG-inducible expression vector to create a genomic (g)DNA library. The gDNA library was transformed into E.coli and pools of ~30 clones were arrayed in 2xYT+carb in 384 deep well plates. Overnight cultures were diluted, grown to log phase and then protein expression was induced overnight at room temperature (RT). Supernatants were removed and bacteria were transferred in PBS to 384 PCR plates to be heat-killed in a thermocycler. Pools of microbiota-reactive TCRs were cocultured with the heat-killed bacteria and mutuDC1s overnight. Supernatants from the coculture assay were assessed for the presence of IL-2 by ELISA. If a well was positive for IL-2, individual TCR hybridomas were screened for reactivity against the pooled bacteria. Then the pool-reactive TCRs were cocultured with individual heat-killed bacteria colonies from the pool. Plasmids were isolated from stimulatory bacteria and sent for whole plasmid sequencing. Any protein sequence present in the plasmid insert was searched for protein coding sequences that were scored for H2 Ab binding likelihood. Strong binding peptides were tested for their ability to stimulate in coculture with the TCR hybridoma and mutuDC1s. (B-F) Heat maps of reactivity measured by IL-2 production are shown for (B) Amuris-, (C) Eclo-, (D) Bcae-, (E) Efae- and (F) Bani-reactive TCRs in coculture assays.

bioRxiv preprint doi: https://doi.org/10.64898/2026.09.23.753882; this version posted September 29, 2026. The copyright holder for this preprint (which was not certified by peer review) is the author/funder, who has granted bioRxiv a license to display the preprint in perpetuity. It is made available under a CC-BY 4.0 International license.

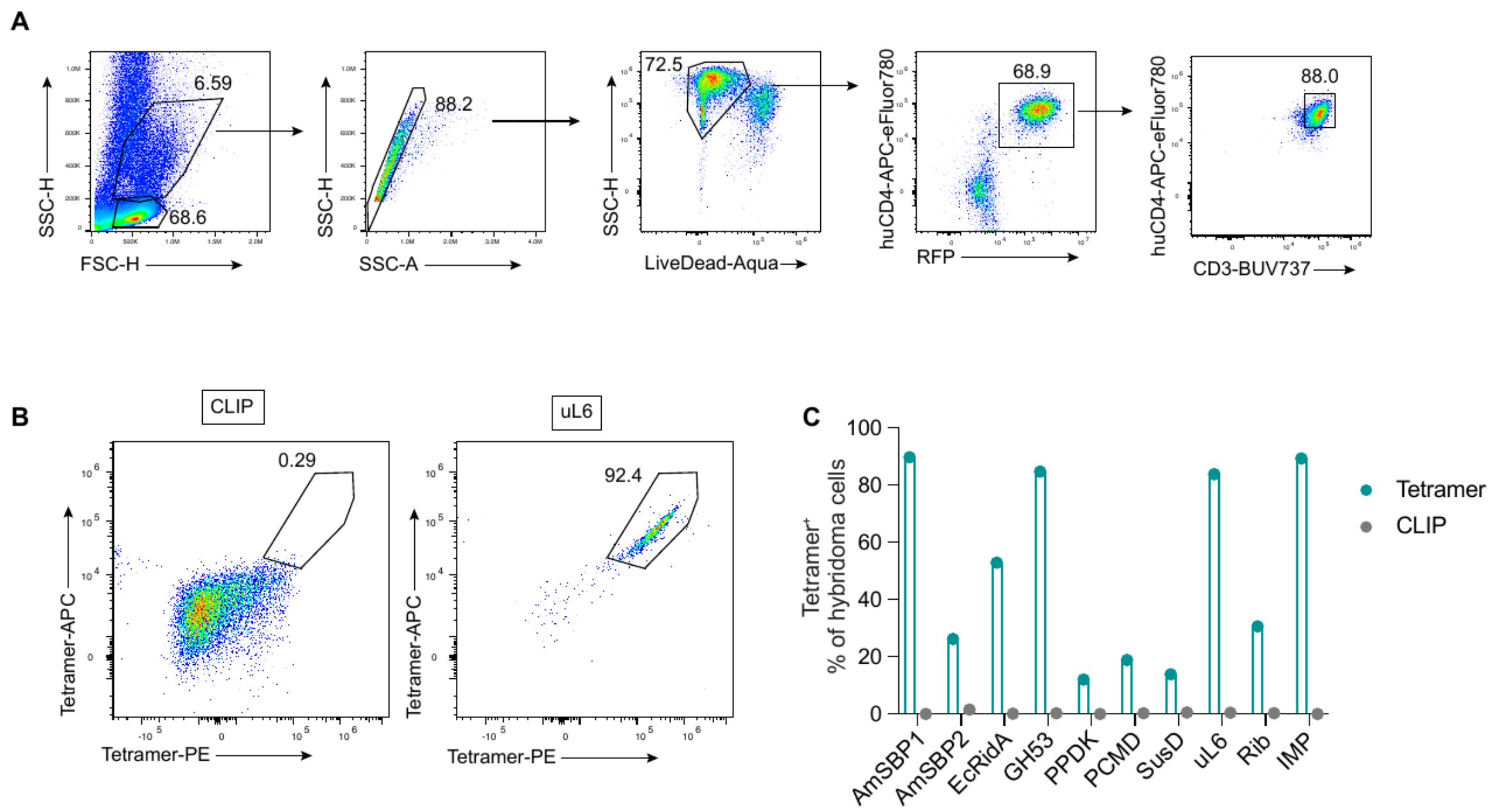

Figure S6. | Validation of MHC class II tetramer specificity. (A) Gating strategy for identifying tetramer-stained hybridoma cells. Hybridomas were gated on FSC-H/SSC-H, singlets (SSC-H/SSC-A), viable cells (LiveDead-Aqua$^-$), huCD4$^+$RFP$^+$, and CD3$^+$huCD4$^+$ prior to tetramer staining. (B) Representative dual-color tetramer staining (Tetramer-PE vs. Tetramer-APC) of the uL6-specific hybridoma, gated on the CD3$^+$huCD4$^+$ population in (A), stained with a CLIP-loaded negative-control tetramer (left) or the cognate uL6 tetramer (right).(C) Quantification of tetramer specificity across all ten epitope-specific hybridomas. Percentage of hybridoma cells stained by the cognate tetramer (teal) versus the CLIP-loaded negative-control tetramer (gray) for AmSBP1, AmSBP2, EcRidA, GH53, PPDK, PCMD, SusD, uL6, Rib, and IMP.

bioRxiv preprint doi: https://doi.org/10.64898/2026.09.23.753882; this version posted September 29, 2026. The copyright holder for this preprint (which was not certified by peer review) is the author/funder, who has granted bioRxiv a license to display the preprint in perpetuity. It is made available under a CC-BY 4.0 International license.

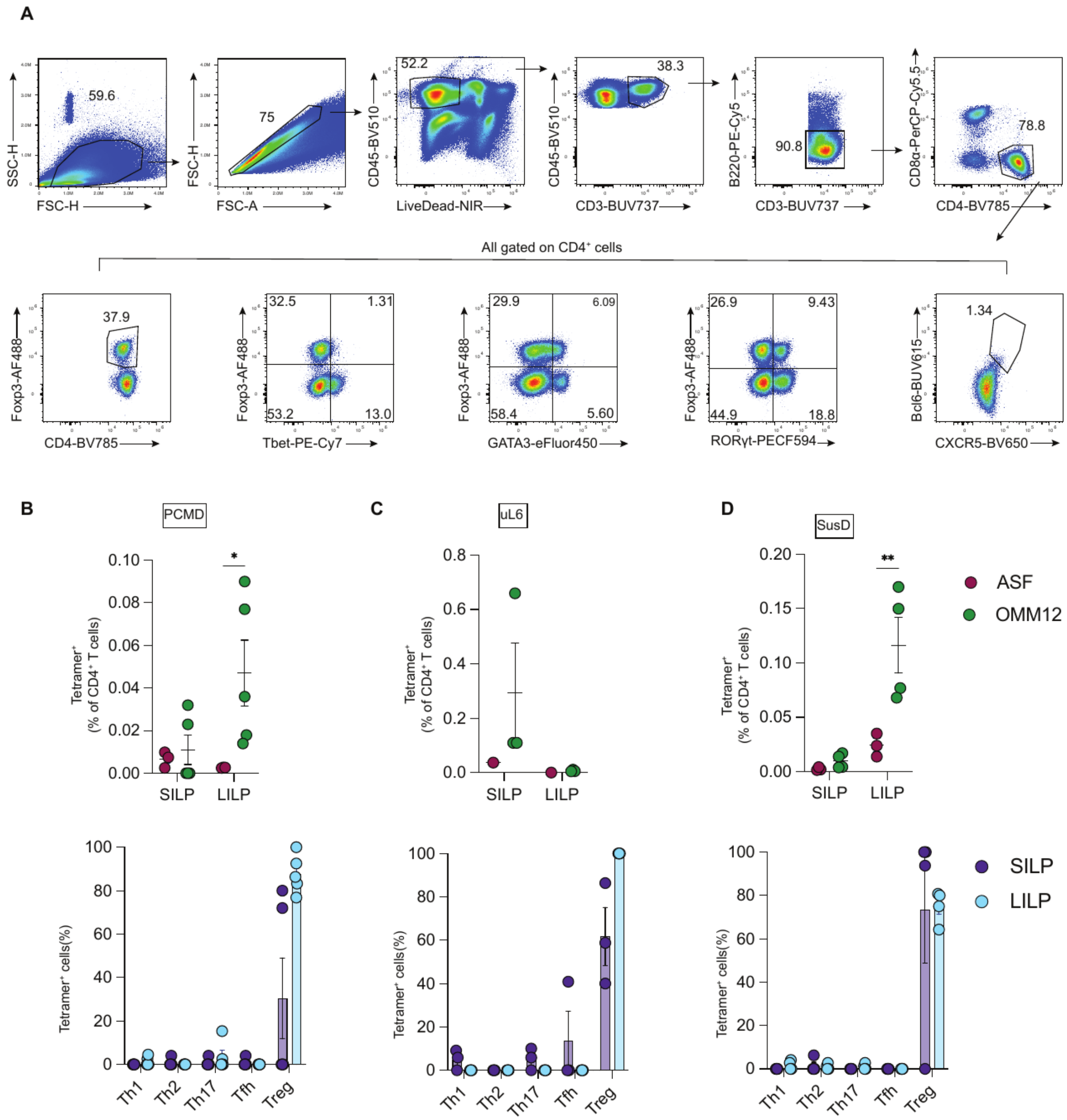

Figure S7. | Gating strategy for identifying antigen-specific CD4⁺ T cells in vivo and validation of Bcae-tetramers individually. (A) Gating strategy for identifying tetramer⁺ CD4⁺ T cell subsets in vivo. Cells were gated on FSC-H/FSC-A, singlets, viable CD45⁺ cells, CD3⁺, B220⁻, and CD4⁺CD8α⁻ T cells. The tetramer⁺ gate and all subset-defining gates (from left to right): Foxp3⁺ (Treg), Tbet⁺Foxp3⁻ (Th1), GATA3⁺Foxp3⁻ (Th2), RORγt⁺Foxp3⁻ (Th17), and Bcl6⁺CXCR5⁺ (Tfh), were drawn on the CD4⁺ T cell population. The Th gates were subsequently mirrored onto the tetramer⁺ gate. (B–D) Frequency of individual Bcae-tetramer⁺ cells among CD4⁺ T cells in the small intestinal (SILP) and large intestinal (LILP) lamina propria of ASF and OMM12 mice (top), and phenotype distribution of tetramer⁺ cells in each tissue (bottom). Each dot represents an individual mouse. Statistical comparisons were not performed for (C) due to insufficient sample size in some groups. For (B,D) tetramer frequency statistical significance was determined by two-way ANOVA with Sidak's multiple comparisons test; *$p < 0.05$, **$p < 0.01$.

bioRxiv preprint doi: https://doi.org/10.64898/2026.09.23.753882; this version posted September 29, 2026. The copyright holder for this preprint (which was not certified by peer review) is the author/funder, who has granted bioRxiv a license to display the preprint in perpetuity. It is made available under a CC-BY 4.0 International license.

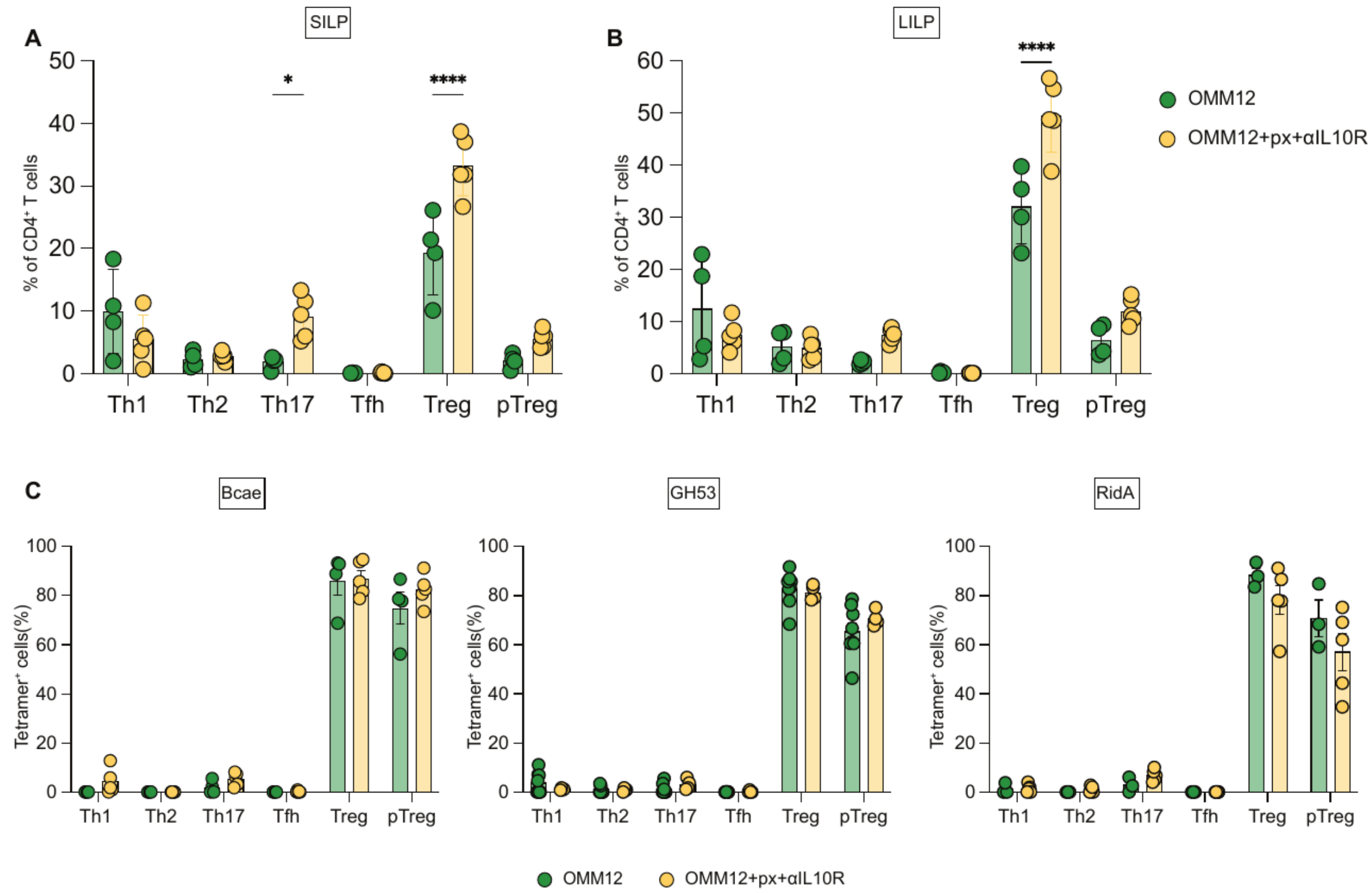

Figure S8. | Characterization of Piroxicam and IL-10R blockade and additional tetramer phenotyping, related to Figure 4. (A, B) Phenotype distribution of endogenous CD4⁺ T cells in the SILP (A) and LILP (B) of OMM12 vs. OMM12+piroxicam+anti-IL-10R treated mice. (C) Phenotype distribution of tetramer⁺ CD4⁺ T cells from the LILP for the pooled Bcae tetramer, GH53, and RidA, comparing OMM12 and OMM12+piroxicam+anti-IL-10R treated mice. For (C) and (D), only mice with tetramer⁺ frequency exceeding the mean + 1 SD of the ASF control frequency were included (see Methods). Data are shown as mean ± SEM. Statistical significance was determined by two-way ANOVA with Sidak's multiple comparisons test; *$p < 0.05$, ****$p < 0.0001$.

bioRxiv preprint doi: https://doi.org/10.64898/2026.09.23.753882; this version posted September 29, 2026. The copyright holder for this preprint (which was not certified by peer review) is the author/funder, who has granted bioRxiv a license to display the preprint in perpetuity. It is made available under a CC-BY 4.0 International license.

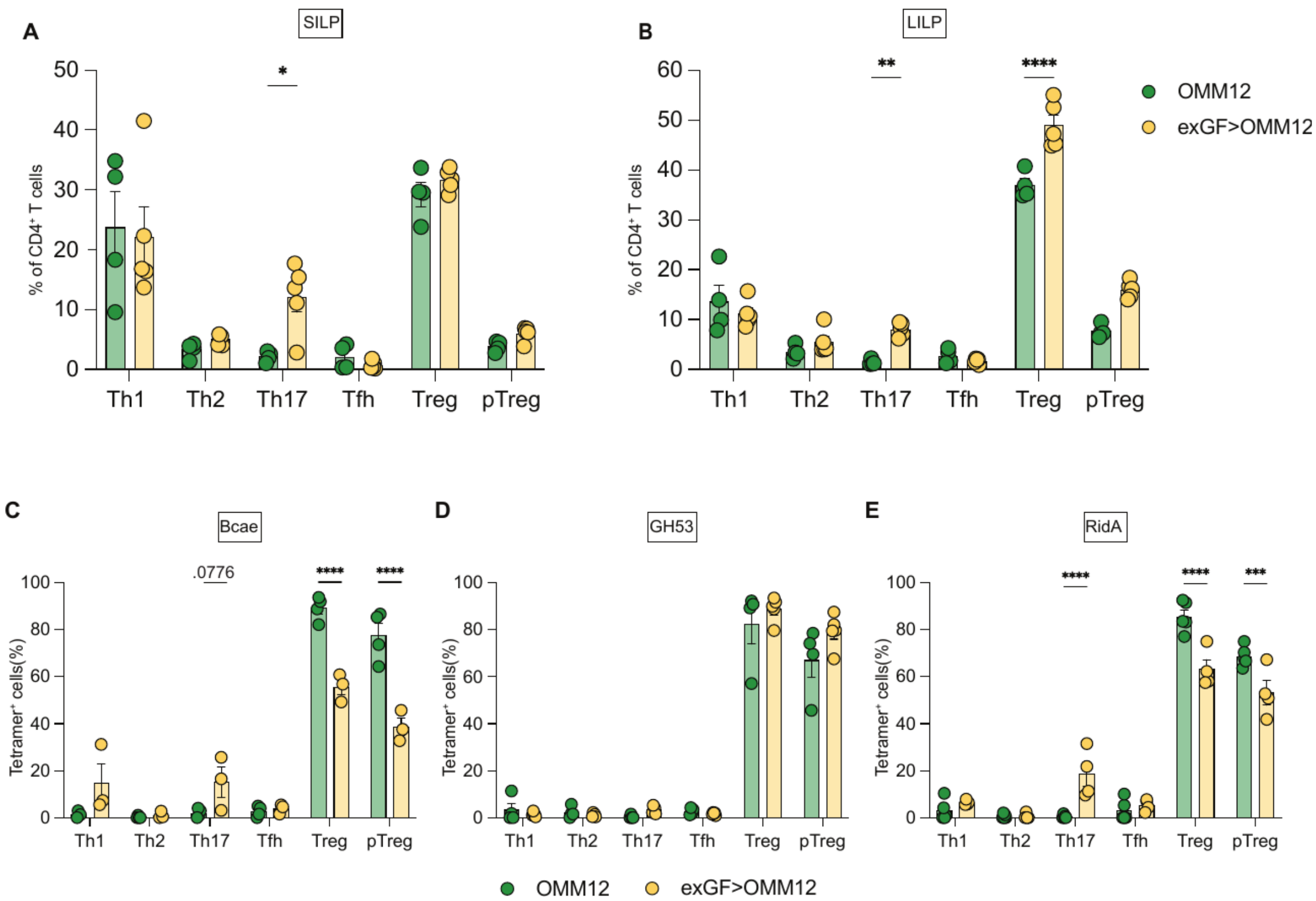

Figure S9. | Horizontal colonization alters antigen-specific T cell phenotype in an epitope-dependent manner, related to Figure 5. (A, B) Phenotype distribution of endogenous CD4⁺ T cells in the SILP (A) and LILP (B) of OMM12 vs. exGF>OMM12 mice. (C–E) Phenotype distribution of tetramer⁺ CD4⁺ T cells from the LILP for the pooled Bcae tetramer (C), GH53 (D), and RidA (E), comparing OMM12 and exGF>OMM12 mice. Only mice with tetramer⁺ frequency exceeding the mean + 1 SD of the ASF (C, D) or GF (E) control frequency were included (see Methods). Data are shown as mean ± SEM. Statistical significance was determined by two-way ANOVA with Sidak's multiple comparisons test; *$p < 0.05$, **$p < 0.01$, ***$p < 0.001$, ****$p < 0.0001$.

bioRxiv preprint doi: https://doi.org/10.64898/2026.09.23.753882; this version posted September 29, 2026. The copyright holder for this preprint (which was not certified by peer review) is the author/funder, who has granted bioRxiv a license to display the preprint in perpetuity. It is made available under a CC-BY 4.0 International license.

SUPPLEMENTAL TABLES

Table S1. | TRAV and TRBV plasmid sequences used in the generation of Sleeping Beauty Transposon-TCR constructs. All mouse TRAV and TRBV sequences that were cloned into the Sleeping Beauty Transposon or pCR2-Blunt II-TOPO vector, respectively. Description of columns is as follows: Variable gene name: Cloned TCR alpha or beta variable gene name, Notes: any differences notes from 10x reference genome, Variable gene sequence = Variable gene sequence in plasmid, Note: Shared plasmid backbone sequences are listed as separate entries with [TRAV] or [TRBV] indicating where the additional TRAV or TRBV sequence is inserted.

Table S2. | All cloned TCR sequences and reactivity metadata. Sequences of all TCRs that were reactive in at least one condition and all information associated with that reactivity. Description of columns is as follows: TCR ID = name of plasmid. Condition = 10x dataset. Clonotype = Cellranger assigned clonotype. TRAV Consensus Used = Cellranger assigned consensus sequence name. TRBV Consensus Used = Cellranger assigned consensus sequence name. Sequence = Nucleotide sequence in the plasmid. TRAV = TRAV name in TCR. CDR3a = amino acid sequence of CDR3a. TRBV = TRBV name in TCR, CDR3b = amino acid sequence of CDR3b, Source = Species of bacteria or other source (i.e. Chow) that the TCR reacts with. (Mmus = self, NE = cloning attempted but not expressed, NR = tested and not reactive under any tested conditions, GFCe = Ceca contents from germ-free mice, OMMCe = Ceca contents from OMM12-colonized mice, underscore used between reactive sources). Protein = Protein name if identified (UNK = Unknown), Peptide = Peptide amino acid sequence if identified (UNK = unknown). gDNA screened = Included in any bacterial genomic DNA library screen. Confidence = Filter for inclusion in any plots of reactivity (LOW = requires followup, HIGH = confirmed in at least 2 coculture experiments, if underscore present then order corresponds to Source). TRAV = TRAV in consensus. TRAJ = TRAJ in consensus. CDR3a (aa) = amino acid sequence of CDR3a. CDR3a nt (contig) = nucleotide sequence of CDR3a. TRBV = TRBV in consensus. TRBJ = TRBJ in consensus. CDR3b (aa) = amino acid sequence of CDR3b. CDR3b nt (contig) = nucleotide sequence of CDR3b. TCRa nt (from construct) = TCRa sequence present in plasmid. TCRa matches contig = If TRUE, TCR construct and consensus match exactly, if FALSE, TCR construct differs from consensus. TCRb nt (from construct) = TCRb sequence present in plasmid. TCRb matches contig = If TRUE, TCR construct and consensus match exactly, if FALSE, TCR construct differs from consensus. Mismatch details = explanation of why a difference exists (e.g. used nearly identical TRAV/TRBV, nucleotide sequence changed to accommodate cloning).
